# Investigating the Role of Interventricular Interdependence in Development of Right Heart Dysfunction During LVAD Support: A Patient-Specific Methods-Based Approach

**DOI:** 10.3389/fphys.2018.00520

**Published:** 2018-05-11

**Authors:** Kevin L. Sack, Yaghoub Dabiri, Thomas Franz, Scott D. Solomon, Daniel Burkhoff, Julius M. Guccione

**Affiliations:** ^1^Division of Biomedical Engineering, Department of Human Biology, University of Cape Town, Cape Town, South Africa; ^2^Department of Surgery, University of California, San Francisco, San Francisco, CA, United States; ^3^Bioengineering Science Research Group, Engineering Sciences, Faculty of Engineering and the Environment, University of Southampton, Southampton, United Kingdom; ^4^Department of Medicine, Brigham and Women's Hospital, Boston, MA, United States; ^5^Cardiovascular Research Foundation, New York, NY, United States

**Keywords:** heart failure, finite element method, realistic simulation, ventricular function, right ventricle, ventricular assist device, mechanical circulatory support

## Abstract

Predictive computation models offer the potential to uncover the mechanisms of treatments whose actions cannot be easily determined by experimental or imaging techniques. This is particularly relevant for investigating left ventricular mechanical assistance, a therapy for end-stage heart failure, which is increasingly used as more than just a bridge-to-transplant therapy. The high incidence of right ventricular failure following left ventricular assistance reflects an undesired consequence of treatment, which has been hypothesized to be related to the mechanical interdependence between the two ventricles. To investigate the implication of this interdependence specifically in the setting of left ventricular assistance device (LVAD) support, we introduce a patient-specific finite-element model of dilated chronic heart failure. The model geometry and material parameters were calibrated using patient-specific clinical data, producing a mechanical surrogate of the failing *in vivo* heart that models its dynamic strain and stress throughout the cardiac cycle. The model of the heart was coupled to lumped-parameter circulatory systems to simulate realistic ventricular loading conditions. Finally, the impact of ventricular assistance was investigated by incorporating a pump with pressure-flow characteristics of an LVAD (HeartMate II™ operating between 8 and 12 k RPM) in parallel to the left ventricle. This allowed us to investigate the mechanical impact of acute left ventricular assistance at multiple operating-speeds on right ventricular mechanics and septal wall motion. Our findings show that left ventricular assistance reduces myofiber stress in the left ventricle and, to a lesser extent, right ventricle free wall, while increasing leftward septal-shift with increased operating-speeds. These effects were achieved with secondary, potentially negative effects on the interventricular septum which showed that support from LVADs, introduces unnatural bending of the septum and with it, increased localized stress regions. Left ventricular assistance unloads the left ventricle significantly and shifts the right ventricular pressure-volume-loop toward larger volumes and higher pressures; a consequence of left-to-right ventricular interactions and a leftward septal shift. The methods and results described in the present study are a meaningful advancement of computational efforts to investigate heart-failure therapies *in silico* and illustrate the potential of computational models to aid understanding of complex mechanical and hemodynamic effects of new therapies.

## Introduction

In view of the growing number and dismal prognosis of patients with end-stage heart failure, interest in emerging mechanical therapies such as left ventricular assistance devices (LVADs) has intensified. LVADs are used as a bridge to transplant, bridge to decision, destination therapy and, increasingly, as a bridge to recovery. The latter is fueled by the nearly ubiquitous demonstration that left ventricular (LV) unloading provided by LVADs causes reverse remodeling and, in a small percentage of patients, induces myocardial recovery to the point where devices can be explanted (Wohlschlaeger et al., 2005; Birks et al., 2006; Burkhoff et al., 2006; Lampropulos et al., 2014; McIlvennan et al., 2014; Topkara et al., 2016). The introduction of smaller, partial (i.e., low) flow LVADs designed to be implanted at an earlier stage of disease severity has broadened the potential applicability to a currently underserved and large patient population (Mohite et al., 2014; Sabashnikov et al., 2014; Sack et al., 2016).

Among the remaining adverse effects that impact negatively on long-term morbidity and mortality of LVAD patients is right heart failure (Grant et al., 2012; Hayek et al., 2014; Rich et al., 2017). Ten to thirty percent of LVAD patients develop right ventricular (RV) failure (Kavarana et al., 2002; Dang et al., 2006; Kormos et al., 2010; Baumwol et al., 2011; Argiriou et al., 2014) requiring either prolonged use of inotropic therapy or the need for temporary or long-term RV mechanical circulatory support. RV failure is associated with elevated central venous pressure (CVP), which adversely affects renal, hepatic, and gastorintestinal function, and results in LV underfilling that reduces LVAD flow.

LVAD support influences RV function in several ways; some beneficial and some detrimental. On the one hand, LVAD-induced unloading leads directly to a reduction of pulmonary capillary wedge pressure. This pressure accounts for a large part of the mechanical afterload on the RV so its reduction can favorably impact the ability of the RV to eject blood and maintain a normal CVP. On the other hand, LVADs increase systemic blood flow, which can cause a volume overload on the RV. Additionally, LV unloading can have a detrimental effect on RV function due to interventricular interactions (Kavarana et al., 2002; Küçüker et al., 2004; Dang et al., 2006; Maeder et al., 2009). This latter effect is a consequence of the interdependence of RV and LV pressure generation mediated by the interventricular septum. It is well-known that as much as 30% of RV pressure generation is due to LV pressure generation and that the position of the interventricular septum can influence RV function (Slater et al., 1997). Thus, LVADs can impact RV function because the RV and LV are two pumps working functionally in series within the circulation, are anatomically arranged in parallel with each other, and share a common wall.

Although the potential implications of ventricular interactions on RV function during LVAD support are well-appreciated, no study has yet proven, in any setting, that LV unloading and septal shift can actually lead to RV failure. This is because it is physically impossible to separate the hemodynamic effects of the serial and parallel contributions of RV-LV interactions in a patient or even in experimental preclinical studies.

Computational modeling is well-suited to investigate and elucidate the individual contributions of these primary hemodynamic factors. However, research efforts have been impeded by the substantial complexities involved in coupling a simulated circulatory system with geometrically realistic models of the heart. Only recently have computational models had the necessary sophistication to model this coupled behavior (e.g., Kerckhoffs et al., 2007; Lim et al., 2012; Baillargeon et al., 2014; Sack et al., 2016). Consequently, very limited research has been undertaken to explore the effect of LVAD function on ventricular mechanics, and no study has investigated the important issue of right heart failure. Such research has significant practical implications since current guidelines for the care of LVAD patients recommend that LVAD speeds be adjusted to ensure that the interventricular septum is not leftward shifted. This recommendation is based on expert opinion, not on any physiological or clinical evidence.

We previously created a model of a failing LV supported with partial LV assistance in a four-chamber generic heart model (Sack et al., 2016). In the present study we modify this representation to include a biventricular model of a patient with dilated cardiomyopathy. LVAD therapy is then simulated using realistic pressure-flow relations of a commonly used LVAD, allowing us to capture assisted flow for device operation over a broad range of rotational speeds (RPMs). By analyzing the resulting changes in LV pressure generation, total blood flow, myocardial stress, and septal wall motion, we quantified the relative influences of these factors on RV function. The specific purpose of this paper is to describe the mathematical methods and general behavior of this model of the failing heart during different degrees of LVAD-induced LV unloading.

## Methods

Our cardiac modeling methods have been described extensively in previous studies (Baillargeon et al., 2014, 2015; Sack et al., 2016). Here, we present a brief overview of these established methods, with an additional focus on recent developments and methods that are critical for the current study.

### Patient data

*In vivo* cardiac magnetic resonance (MR) data sets were obtained as part of the Aliskiren Study in Post-MI Patients to Reduce Remodeling (ASPIRE) trial (Solomon et al., 2011). Individual patients provided informed consent and anonymized data were sent to a core laboratory for analysis.

### Geometric considerations

**Figure 1:**
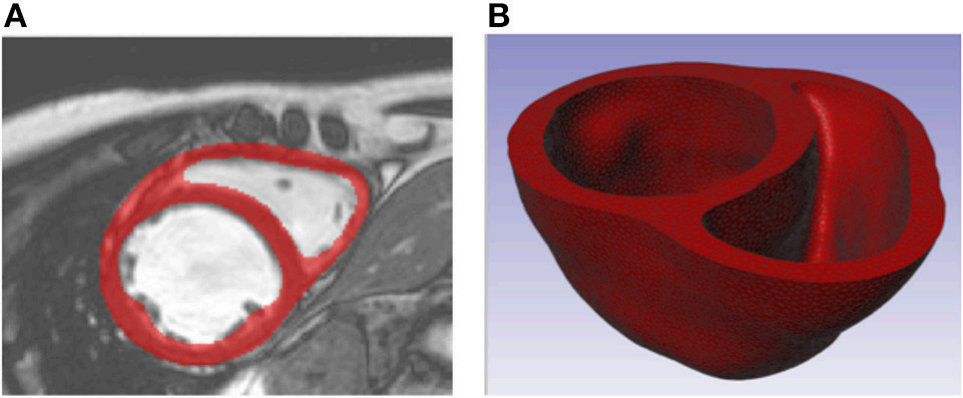
**(A)** Short axis MR image of patient with dilated chronic heart failure overlaid with the segmentation of the myocardium. **(B)** Truncated biventricular geometry extracted from segmentation and meshed using tetrahedral elements.

For one patient with dilated cardiomyopathy, the MR data sets (1.25 × 1.24 × 10 mm spatial resolution) were imported and processed in Simpleware ScanIP (Synopsys, Mountain View, USA). Geometrically detailed segmentations of the LV and RV were created relying on a combination of well-established techniques, including region growing, level-set thresholding, and morphological smoothing (Vadakkumpadan et al., 2010; Setarehdan and Singh, 2012). The biventricular structure was truncated at the base and illustrations of the image data, segmentation, and Finite Element (FE) mesh construction are presented in Figure 1.

We introduced prolate spheroidal coordinates (Lombaert et al., 2012; Toussaint et al., 2013) into the image-coordinate space aligned with the long axis of the LV. The prolate spheroidal coordinates were used to describe myofiber orientations using a rule-based approach. Based on previous computational studies (Genet et al., 2014), histological studies (Streeter et al., 1969), and diffusion tensor MR studies (Lombaert et al., 2011), we assumed that the myofiber orientation could be represented through a linearly varying helix angle from −60° on the epicardium to +60° on the endocardium. This was assigned to each material point in the model through a custom MATLAB (The MathWorks, Inc., Natick, Massachusetts, United States) script that specifies myofiber orientation by rotating the local circumferential unit vector by the helix angle in the circumferential-longitudinal plane. The same fiber description from endocardium to epicardium was applied to the LV free wall, septal wall, and RV free wall as other studies typically assume (Goktepe et al., 2011; Wenk et al., 2012; Wong and Kuhl, 2014).

While multiple models and explanations of sheet structure exist (Gilbert et al., 2007), for simplicity we define the sheet directions to be normal with epicardial and endocardial surfaces (i.e., normal with the circumferential-longitudinal plane in which the fiber direction resides). This assumption is relatively reasonable when considering the macroscopically visible cleavage planes observed experimentally (LeGrice et al., 2001; Chen et al., 2005) and is in line with other computational studies (Bovendeerd et al., 1994; Goktepe et al., 2011).

Regarding boundary conditions, the base of the biventricular structure (plane of truncation) was fixed in the longitudinal direction. Furthermore, the nodes on the endocardial annulus were constrained by coupling the average translation and rotation of the nodes to a fixed point in space located at the annulus center. This prevents rigid body rotation while allowing the annulus relative motion to inflate and contract during the cardiac cycle.

### Constitutive law and parameter estimation

The passive material response of the cardiac tissue uses an anisotropic hyperelastic formulation proposed by Holzapfel and Ogden (Holzapfel and Ogden, 2009). The isochoric and volumetric responses are governed by the strain energy potentials in Equations (1–2)

(1) $$\begin{array}{l} \Psi_{iso}=\frac{a}{2b}e^{b(I_{1}-3)}+\;\sum_{i\;=\;f,s}\frac{a_{i}}{2b_{i}}\left\{e^{b_{i}{\left(I_{4i}-1\right)}^{2}}-1\right\}\\ \;+\;\frac{a_{fs}}{2b_{fs}}\left\{e^{b_{fs}{\left(I_{8fs}\right)}^{2}}-1\right\}, \end{array}$$

(2) $$\begin{array}{l} \Psi_{vol}=\frac{1}{D}\left(\frac{J^{2}-1}{2}-\ln\left(J\right)\right). \end{array}$$

Equation (1) is defined through eight material parameters *a, b, a*_*f*_*, b*_*f*_*, a*_*s*_*, b*_*s*_*, a*_*fs*_*, b*_*fs*_ and four strain invariants *I*_1_, *I*_4*f*_, *I*_4*s*_, and *I*_8*fs*_. These strain invariants are derived from the isochoric right Cauchy-Green tensor,

(3) $$\begin{array}{l} \bar{C}=\;{\bar{F}}^{T}\;\bar{F}=J^{-2/3}C=J^{-2/3}F^{T}F \end{array}$$

where **F** is the deformation gradient, *J* is the determinant of the deformation gradient, *J* = det(***F***) and $\bar{F}$ is the isochoric part of the deformation gradient such that

(4) $$\begin{array}{l} \bar{F}=J^{-1/3}F\text{ and det}\left(\bar{F}\right)=1. \end{array}$$

The expression of these strain invariants can now be defined as:

(5) $$\begin{array}{l} I_{1}=tr\left(\bar{C}\right),\;I_{4f}=f_{0}\cdot \left(\bar{C}f_{0}\right),I_{4s}=s_{0}\cdot \left(\bar{C}s_{0}\right),I_{8fs}=f_{0}\cdot \left(\bar{C}s_{0}\right) \end{array}$$

Where *f*_0_ and *s*_0_ are orthogonal vectors in the fiber and sheet direction in the reference configuration. Equation (2) is defined through *J* and a penalty term *D*, which is a multiple of the bulk modulus (*D* = 2/*K*). For deformation that perfectly preserves volume, *J* = 1.

This passive material model, Equations (1–2), ensures that the material exhibits the well-documented exponential and anisotropic response to strain (Demer and Yin, 1983; Hunter et al., 1998; Dokos et al., 2002) while enforcing incompressibility.

The description of our time-varying elastance model of active force development (Guccione and McCulloch, 1993) is specified as:

(6) $$\begin{array}{l} T_{a}\left(t,l\right)=T_{MAX}\frac{Ca_{0}^{2}}{Ca_{0}^{2}+ECa_{50}^{2}\left(l\right)}\frac{\left(1-\cos\left(\omega \left(t,l\right)\right)\right)}{2} \end{array}$$

where *T*_*max*_, the maximum allowable active tension, is multiplied with a term governing the calcium concentration, and a term governing the timing of contraction. Both terms depend on sarcomere length *l*, which in turn depends on the strain in the fiber direction. The active tension generated from this representation conforms well with experimental studies (Guccione and McCulloch, 1993) and captures length-dependent effects such as Frank Starling's Law (Holmes et al., 2002; Solaro, 2007). Further detail of the active tension law is provided in the Appendix for the interested reader.

We consider the total Cauchy stress to be an additive contribution of passive and active components. The passive Cauchy stress, **σ_*p*_**, is given by **σ_*p*_** = 2*J*^−1^ ***F***(**∂Ψ**/**∂*C***)***F^T^***. We consider an active contractile stress in the fiber direction, resulting in a total Cauchy stress in the fiber direction, (i.e., myofiber stress) by combining this with to the passive stress state in this direction (**σ**_***pf***_):

(7) $$\begin{array}{l} \sigma_{f}=\sigma_{pf}\;+T_{a\;}f⊗f \end{array}$$

Biaxial investigations on actively contracting rabbit myocardium revealed significant stress development in the cross-fiber direction that could not be completely attributed to myofiber dispersion or deformation effects (Lin and Yin, 1998). This has motivated computational efforts to consider a proportion of the active stress developed in the myofiber direction to be transferred onto the stress in the sheet direction by a scalar *n*_*s*_ ∈ (0, 1), such that the total Cauchy stress in the sheet direction is:

(8) $$\begin{array}{l} \sigma_{s}\;=\;\sigma_{ps}\;+n_{s}\;T_{a\;}s⊗s \end{array}$$

### Material parameter estimation

Full records of the patient who was used in our heart model were unavailable; therefore, we relied on clinical input to provide representative functional targets for volume and pressures. To this end, the dilated failing heart used in this study was assumed to have an end-diastolic volume (EDV) of 254 ml and an end-systolic volume (ESV) of 224 ml. End-diastolic pressure (EDP) and end-systolic pressure (ESP) were assumed to be 23 and 86 mmHg, respectively. These classify the patient with a severely dilated LV (>200 ml), elevated EDP (>16 mmHg, Paulus et al., 2007 and 23 mmHg, Mielniczuk et al., 2007) and severely reduced ejection fraction (EF = 1− ESV/EDV = 12%) i.e., <35% (McMurray et al., 2012; Mann et al., 2014).

The material parameters *a, b, a*_*f*_*, b*_*f*_*, a*_*s*_*, b*_*s*_*, a*_*fs*_*, b*_*fs*_ were found through optimization techniques relying on two stages of determination. Initial values were determined from the calibration of normal myocardium specimen samples to experimental tri-axial shear data of human myocardium (Sommer et al., 2015). Calibration was performed using ABAQUS as the forward solver, whereby *in silico* cubes of myocardium with dimensions matching those of the study of interest were meshed into a uniform 27 linear hex-element mesh. Shearing was executed by specifying the translational displacement of a specified cube face, while enforcing zero displacement boundary conditions on the opposite cube face. The optimization was performed in MATLAB using a non-linear least-square optimization routine.

To capture patient-specific material parameters of our failing human heart, a second stage of “scaling” was needed. Here, linear (*a, a*_*f*_*, a*_*s*_, *and a*_*fs*_) and exponential (*b, b*_*f*_*, b*_*s*_*, b*_*fs*_) terms were subject to uniform scaling by parameters *A* and *B*, a scalar and an exponential multiplier, respectively. These values were found by minimizing the error between the *in silico* diastolic PV course resulting from loading the LV of the FE model to the analytical Klotz curve (Klotz et al., 2006), starting from the unloaded LV volume V_0_ until the EDV was reached at the specified end-diastolic pressure (EDP). Material parameters were calibrated using ABAQUS as the forward solver, and an in-house PYTHON script containing the sequential least squares programming (SLSQP) optimization algorithm (Jones et al., 2001). These passive parameters expressed in Equations (1) were identified by minimizing the error between the *in silico* diastolic PV curve and the analytical Klotz curve.

Once passive parameters were found, the active parameter T_MAX_ was identified by minimizing the error between predicted and specified stroke volume and assuming that 25% of active tension was transferred in the sheet direction (i.e., *n*_*s*_ = 0.25). Calibrated material parameters are presented in Table 1.

**Table 1:** Calibrated material parameters.

**Parameters**	***a***	***b***	***a_*f*_***	***b_*f*_***	***a_*s*_***	***b_*s*_***	***a_*fs*_***	***b_*f*_*_s_**	**T_MAX_**
	**(kPa)**		**(kPa)**		**(kPa)**		**(kPa)**		**(kPa)**
	8.41	30.32	27.72	58.18	3.85	50.44	2.26	12.42	170.0

### Coupled circulatory system and LVAD support

**Figure 2:**
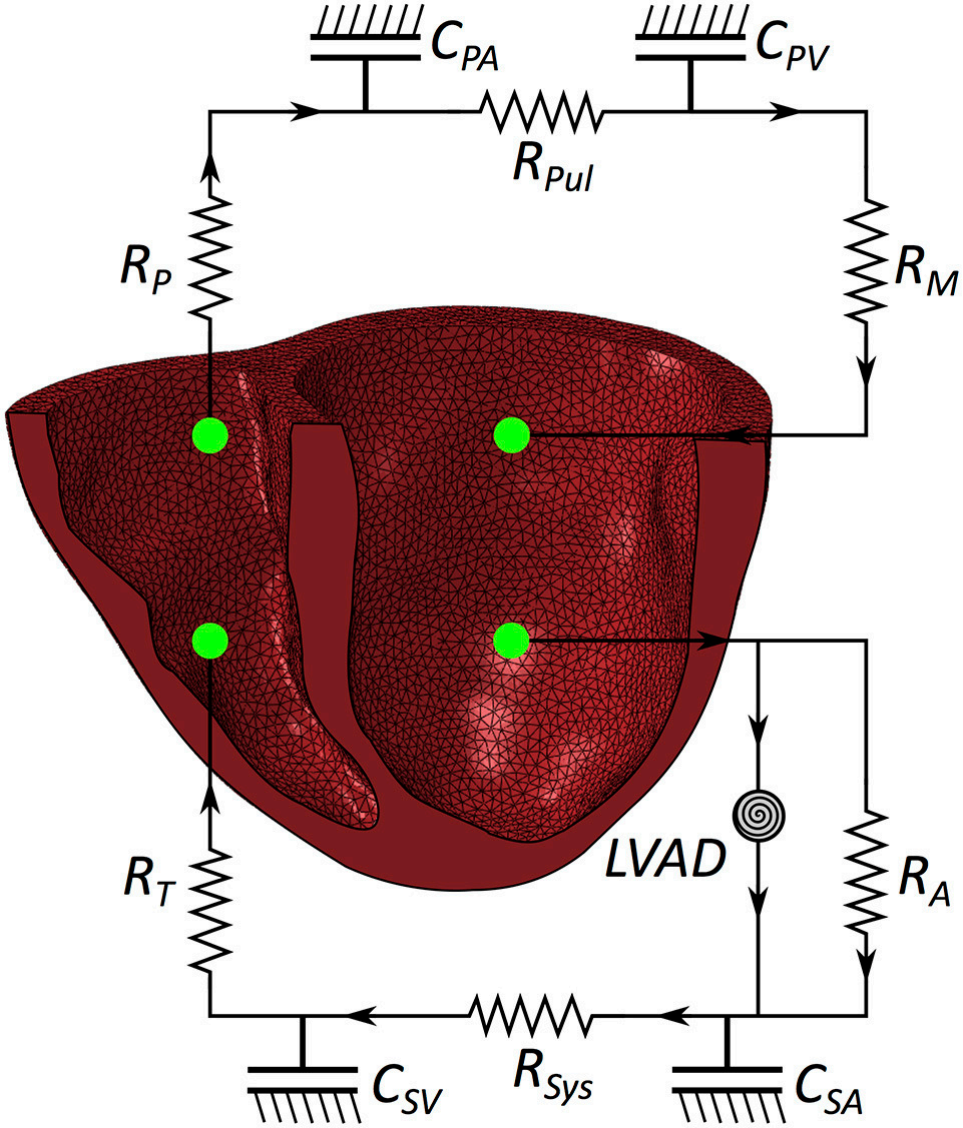
Schematic of the patient-specific biventricular structure coupled with the circulatory system and LVAD. R_M_ is mitral valve resistance, R_A_ is aortic valve resistance, C_SA_ is systemic arterial compliance, R_SYS_ is systemic arterial resistance, C_SV_ is systemic venous compliance, R_T_ is tricuspid valve resistance, R_P_ is pulmonary valve resistance, C_PA_ is pulmonary arterial compliance, R_PUL_ is pulmonary arterial resistance, C_PV_ is pulmonary venous compliance.

The FE model of the heart was coupled to lumped models of the pulmonary and systemic circulatory systems used in our previous study (Sack et al., 2016). A small modification was introduced to separate the pulmonary circuit into venous and arterial components. Collectively, this captures fluid exchanges between the systemic circuit, the heart and the pulmonary circuit. A schematic diagram outlining the fluid connections between the patient-specific biventricular structure and the lumped circulatory system with the LVAD is presented in Figure 2; all parameters relating to the lumped model are presented in the Appendix in Table A2.

Two further changes are present in the model used in this study compared with the model used in our prior study (Sack et al., 2016). First, the mechanical heart is replaced with a patient-specific biventricular structure (as detailed in previous sections).

**Figure 3:**
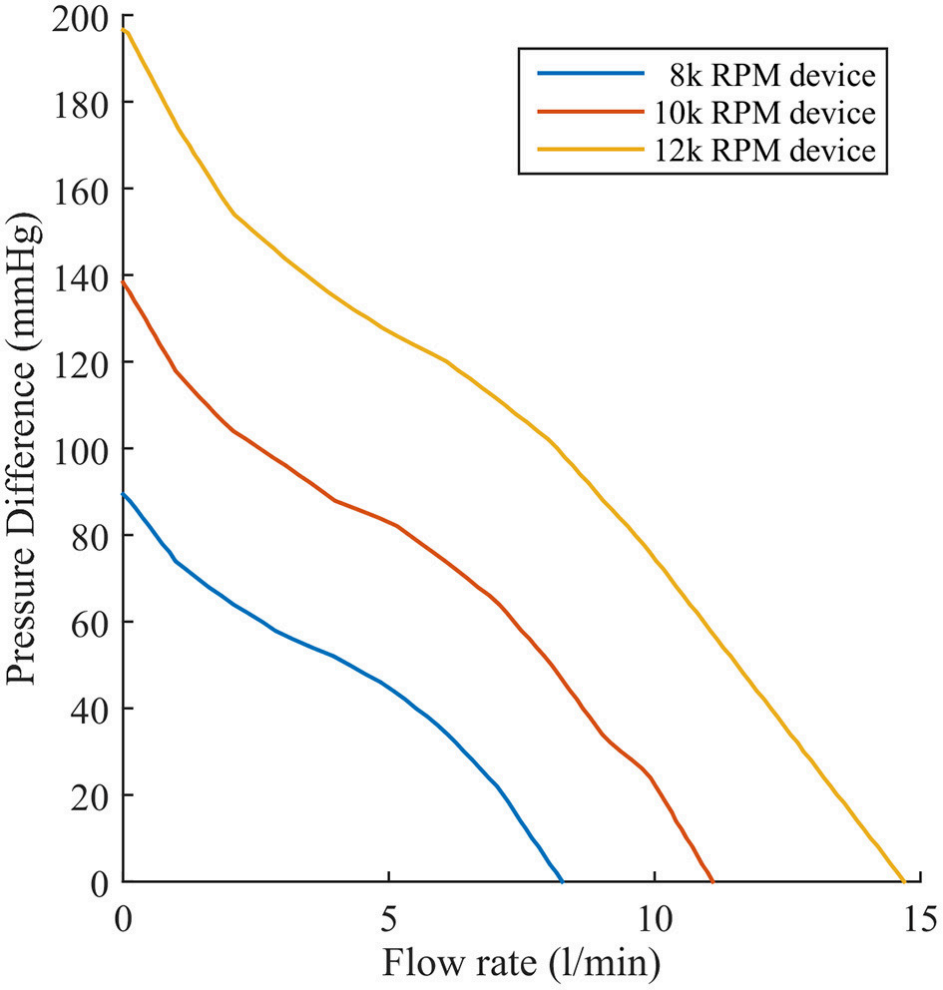
Experimentally recorded flow rates of the Heartmate II™ LVAD operating at 8, 10, and 12 k RPM as a function of pressure difference.

The second change is that a far more complex and realistic representation of LVAD flow is included. We simulated the effect of a Heartmate II ™ LVAD device operating at device speeds of 8, 9, 10, 11, and 12 k RPM. Each speed has a flow rate profile that is dependent on the pressure difference (dP) between the inflow and outflow cavities to which the pump connects. Experimental datasets specifying flow rates for a range of dP between 0 and 200 mmHg at discrete intervals of 2 mmHg were incorporated in the simulated flow profile for each operating speed. Flow rates were interpolated and extrapolated linearly between discrete values to account for a continuous flow-rate description for any dP the model may encounter. This is illustrated in Figure 3 for three LVAD operating speeds (8, 10, and 12 k RPM).

### Experimental design

For this study, cardiac function was simulated for a patient with chronic heart failure. LVAD therapy was introduced by simulating the effect of a HeartMate II operating at speeds ranging from 8–12 k RPM in 1 k RPM increments. Pressure and volume measurements of the ventricular chambers were recorded and compared to quantify ventricular loading and output performance. Myofiber stress was recorded and quantified for each simulation and compared to analyze the efficacy of treatment in the LV and potential harm of treatment to the septal wall and RV. Stress data in this study are expressed as mean ± standard of deviation (*SD*) unless otherwise stated. The differences between results were evaluated using analysis of variance (ANOVA) with differences considered statistically significant with *p* < 0.05. Time points in the cardiac cycle, such as end diastole, were defined for the untreated case and compared to the time points in simulations with LVAD support. End diastole and end systole, for each ventricle, were identified from the pressure-volume curves as the points immediately preceding isovolumetric contraction and relaxation respectively.

## Results

The model of chronic heart failure without LVAD support represents a critical patient with advanced heart failure. The LV is substantially overloaded at end diastole with an EDV of 254 ml, an EDP of 23 mmHg, and an LV EF of 12%. The support introduced through LVAD operation improves these functional metrics: the diastolic loading of the LV decreases and the LV EF increases as the RPM of the device increases (i.e., increased support), as shown in Table 2. These LV benefits occur simultaneously with increases in the RV loading, seen by the rise in RV EDV (Table 2) and EDP which increases from 20.8 mmHg (untreated) to 27.9 mmHg (LVAD operating at 12 k RPM), as the mean central venous pressure rises (Table 2). Although the RV EF initially increases with LVAD operation, it reaches a peak functional value with LVAD support at 11 k RPM, after which it starts to decline.

**Table 2:** Functional metrics in the left and right ventricles.

	**LV EDV**	**PCWP**	**RV EDV**	**CVP**	**LV EF**	**RV EF**	**CO**
	**(ml)**	**(mmHg)**	**(ml)**	**(mmHg)**	**(%)**	**(%)**	**(L/min)**
No intervention	253.9	39.8	165.0	20.8	11.9	16.3	1.82
LVAD 8 k RPM	245.5	39.0	169.5	21.7	12.8	18.5	1.88
LVAD 9 k RPM	238.7	38.3	173.3	22.5	14.5	19.9	2.07
LVAD 10 k RPM	227.8	36.9	178.0	23.6	16.5	21.1	2.25
LVAD 11 k RPM	212.9	34.8	182.0	25.2	18.4	21.5	2.34
LVAD 12 k RPM	190.2	31.7	184.5	27.1	18.5	19.1	2.11

*LV, left ventricle; RV, right ventricle; EDV, end-diastolic volume; EF, ejection fraction; PCWP, pulmonary capillary wedge pressure; CVP, central venous pressure; CO, cardiac output*.

**Figure 4:**
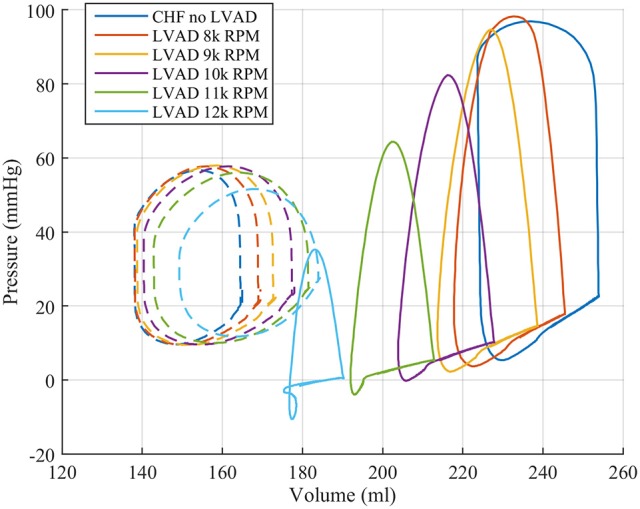
Pressure volume loops for the LV (solid lines) and RV (dashed lines) from the FE model study.

**Figure 5:**
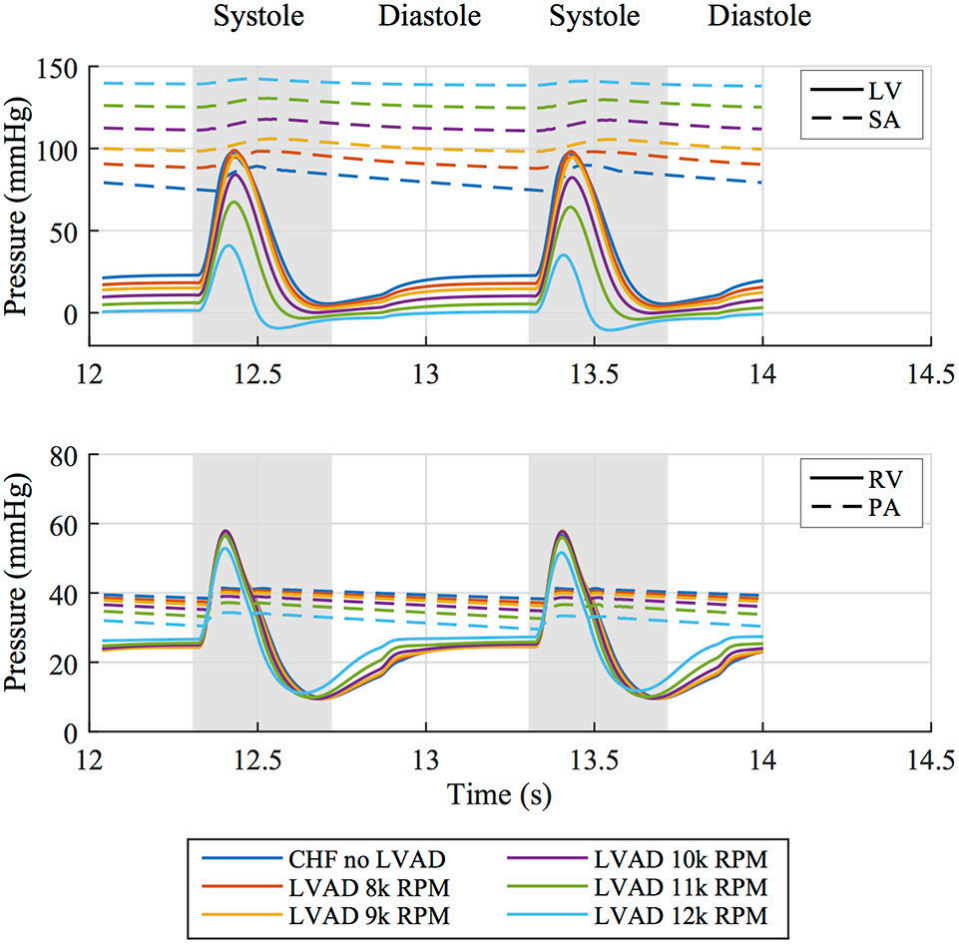
**(Top)** Pressure tracings of the LV and systemic arterial compliance (SA) over two cardiac cycles for all simulated cases. **(Bottom)** Pressure tracings of the RV and pulmonary arterial compliance (PA) over two cardiac cycles for all simulated cases.

These functional changes are also captured in pressure-volume loops of each chamber, as shown in Figure 4. These curves illustrate the reduction of LV EDP, which ranged from 17.9 to 0.5 mmHg, with the simulated LVAD operating between 8 and 12 k RPM. As LVAD flow increases, the LV PV-loop becomes more triangular, indicative of the device's effect during the normally isovolumetric periods of the cardiac cycle. As the loop shifted leftward toward lower volumes, it tracked down a single end-diastolic pressure-volume relationship. As the LV was increasingly unloaded by increases in LVAD speed, the RV PV-loops shifted rightward toward higher volumes and pressures. In addition, the systolic portion of the loops also shifts rightward and peak RV pressure also decreases. These shifts are a consequence of the reductions of LV pressure during both diastole and systole. It is noteworthy that LVAD speeds of up to 12 k increased RV EDP, resulted in excessive reductions of LV volume and, in the case of the 12 k RPM simulation, reduced cardiac output: three distinctive characteristics of right heart failure. Additional pressure tracings of the LV, the systemic arteries (analogous of aortic pressure), the RV and the pulmonary arteries are provided in Figure 5 for two heartbeats.

**Figure 6:**
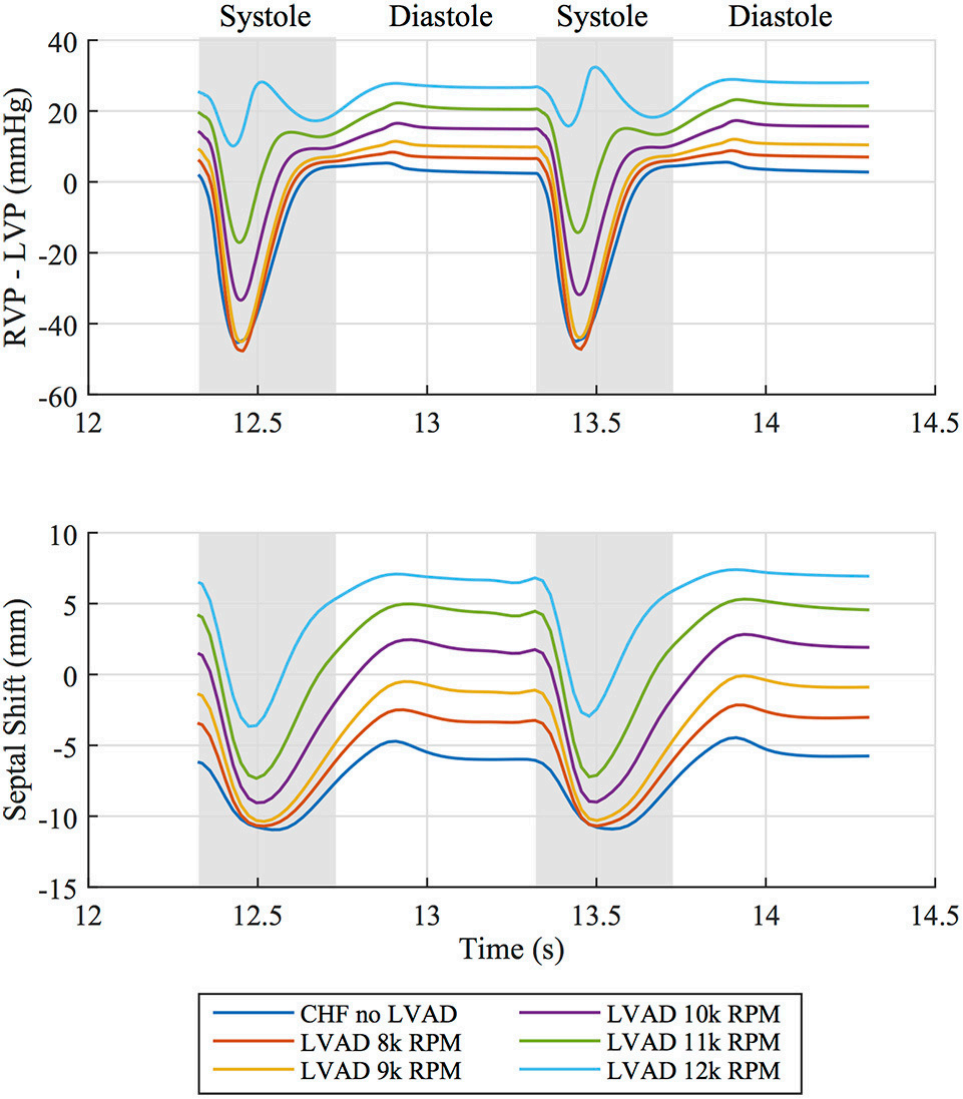
**(Top)** Pressure difference between right ventricular pressure (RVP) and left ventricular pressure (LVP) over two cardiac cycles for all simulated cases. **(Bottom)** Corresponding leftward septal shift over the same time period. Diastolic and systolic portions of the cardiac cycle are labeled.

The interventricular septum is in constant motion throughout the cardiac cycle. We tracked the midpoint motion at the base of the septal wall and quantified the leftward shift of this point in reference to the line through the anterior and posterior LV-septal-RV junctions. This measure of septal shift is positive when the septal wall bulges into the LV cavity, zero when the septal wall forms a straight line and negative when the wall bulges into the RV cavity. A time course of this measure over two heartbeats, shown in Figure 6 alongside the pressure difference between the ventricles, reveals that peak leftward shift occurs during diastolic function and goes from being concave in the unsupported case to convex in the case of simulated LVAD support >10 k RPM. Rightward septal shift coincides with peak systole (and minimum trans-septal pressure) and is driven by the contractile forces with the heart returning the shape to a more “normal” configuration. Septal shift is also shown by plotting displacements in the dynamic beating ventricles of the patient with no intervention and LVAD operating speeds of 8, 10, and 12 k RPM in the Supplementary Animations S1, S2.

The volumetric-averaged myofiber stress (along the local muscle fiber direction) was calculated at end diastole and end systole, and the mean ± *SD* are presented in Table 3 for the RV free wall, the septal wall, and LV free wall separately. Compared to the unsupported CHF case, LV mean myofiber stress is reduced by LVAD support by an order of magnitude at end diastole and end systole (*p* < 0.001). The improvements to RV mean myofiber stress were less substantial, with both end diastole and end systole myofiber stress in the RV remaining relatively unaffected.

**Table 3:** LV and RV myofiber stress results (mean ± *SD*) at end diastole and end systole (*p* < 0.001 for comparisons within each column).

	**LV free wall**		**Septal wall**		**RV free wall**	
	**ED myofiber stress (kPa)**	**ES myofiber stress (kPa)**	**ED myofiber stress (kPa)**	**ES myofiber stress (kPa)**	**ED myofiber stress (kPa)**	**ES myofiber stress (kPa)**
No intervention	13.7 ± 6.8	35.7 ± 15.6	10.0 ± 7.5	29.8 ± 13.8	14.9 ± 10.8	21.4 ± 15.2
LVAD 8 k RPM	10.8 ± 5.7	33.3 ± 14.6	7.9 ± 7.2	27.5 ± 13.2	14.1 ± 10.4	20.8 ± 14.8
LVAD 9 k RPM	8.8 ± 5.0	30.8 ± 13.7	6.8 ± 7.4	25.2 ± 12.6	13.8 ± 10.3	20.4 ± 14.6
LVAD 10 k RPM	6.3 ± 4.2	22.6 ± 10.8	5.8 ± 8.7	17.6 ± 10.9	13.5 ± 10.5	18.9 ± 13.9
LVAD 11 k RPM	3.5 ± 3.8	12.4 ± 8.1	5.1 ± 10.9	8.2 ± 9.9	13.1 ± 10.6	16.5 ± 12.7
LVAD 12 k RPM	1.1 ± 4.2	1.5 ± 12.1	5.1 ± 14.1	−1.0 ± 16.0	13.0 ± 11.2	13.5 ± 11.4

*LV, left ventricle; RV, right ventricle; ED, end diastole; ES, end systole*.

**Figure 7:**
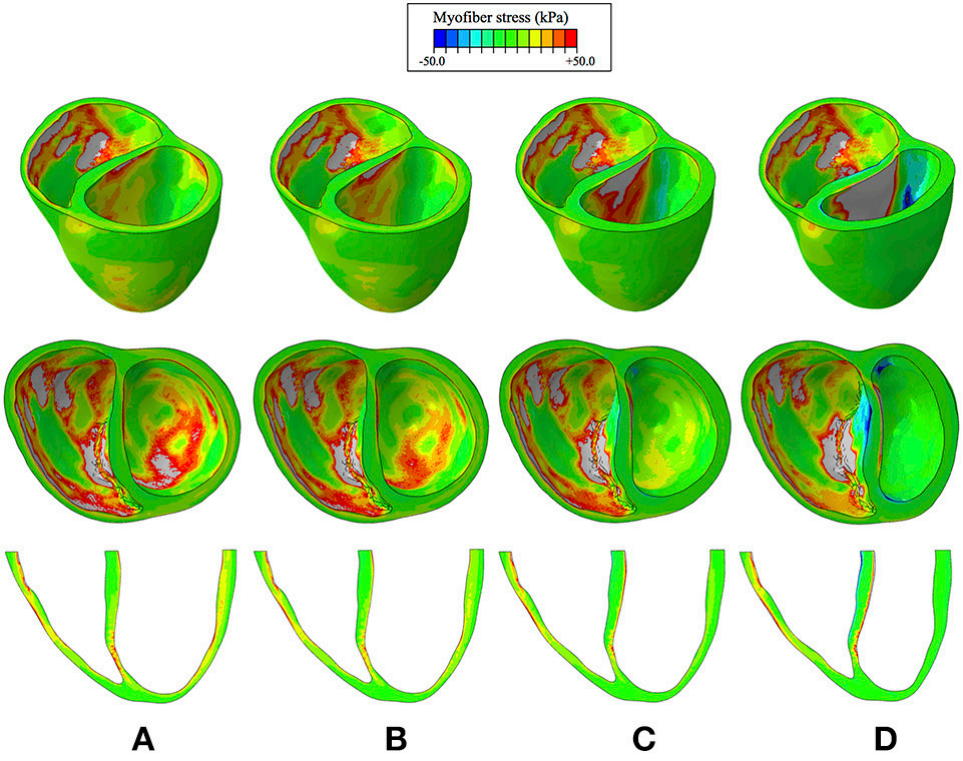
Myofiber stress distributions at end diastole for the cases of **(A)** CHF with no LVAD support, **(B)** CHF with LVAD support running at 8 k RPM, **(C)**, CHF with LVAD support running at 10 k RPM, **(D)** CHF with LVAD support running at 12 k RPM. Top row reveals a predominantly long-axis view of the biventricular structure, the middle row reveals a short-axis view, exposing the ventricular cavities, and the bottom row reveals a long-axis cut plane that bisects the ventricles. Light gray regions correspond to extreme stress values that exceed the threshold of +50 kPa.

Myofiber stress distributions and overall geometry are presented in Figure 7 at end diastole to illustrate stresses and the deformed configuration corresponding to maximum volume loading. These stress distributions reveal geometrically relevant stress characteristics that evolve with increased LVAD operation. The large stress values seen on the LV endocardium (excluding the septal wall) due to volumetric loading at end diastole decrease with LVAD support and appear to dissipate with maximum LVAD operation of 12 k RPM. However, a localized region of tensile (i.e., positive) myofiber stress appears and grows with increased LVAD support on the LV side of the septal wall near the base (Figure 7). Additionally, LVAD operation promotes a localized region of compressive (i.e., negative) myofiber stress on the RV side of the septal wall in the same region.

**Figure 8:**
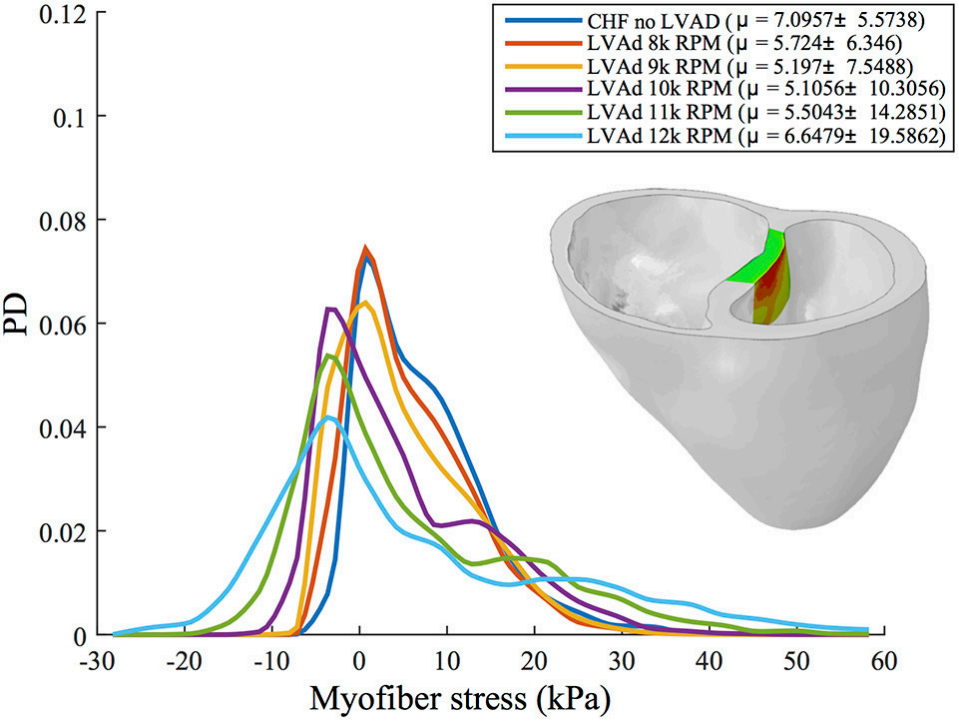
Interpolated histograms of myofiber stress distributions in the region of maximum septal shift (colored region of inlaid illustration) for all simulations of CHF and LVAD operation. Mean ± *SD* values are given in the legend for each case. Histograms are normalized by probability density (PD), i.e., the area under each distribution sums to 1.

A quantitative analysis of myofiber stress distribution in a segmented region of the vulnerable septal wall is presented in Figure 8. Initially (i.e., chronic heart failure with no LVAD support) the myofiber stresses in this region display a mostly Gaussian distribution. At 10 k RPM the myofiber stress distributions in this region begin to display bimodal peaks, which become more exaggerated with increased LVAD support.

## Discussion

We describe a geometrically and physically realistic model of an end-stage failing heart with representative systolic and diastolic myocardial material properties coupled to lumped parameter Windkessel-like models of the pulmonary and systemic circulations. This permitted study of heart mechanics and dynamics under realistic loading conditions i.e., pre-load and afterload of each ventricle. Finally, we simulated the effects of LVAD support by using experimentally recorded pressure-flow characteristics of a commonly used device. The present model represents a significant improvement over our prior modeling efforts (Sack et al., 2016) in that the effects of an LVAD on chronic rather than acute left heart failure were quantified using a patient-specific biventricular geometry and device-specific pressure-flow characteristics, rather than constant flow rates.

**Figure 9:**
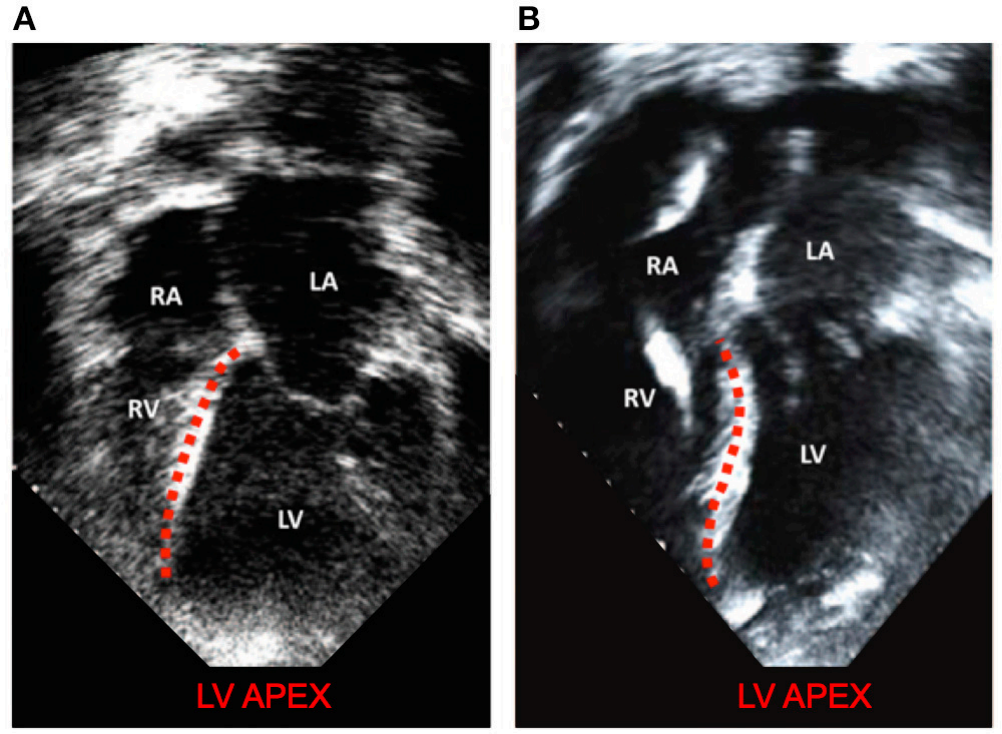
Modified with permission from Topilsky et al. (2011). **(A)** a patient with low RPMs and normal, right shifted interventricular septum (traced out by the red line) compared to **(B)** a patient with markedly left-shifted septum (traced out by the red line). LV, left ventricle; RV, right ventricle; LA, left atrium; RA: right atrium.

This improved model reproduced a wide range of expected, fundamental behaviors of the LV and RV. There were LVAD speed-dependent reductions in LV filling pressure, pressure generation, and a progressive transformation of the PV-loop from trapezoidal shape to triangular shape. This shape transformation is because LVADs are continuous flow pumps that remove volume from the LV throughout the cardiac cycle, thus eliminating isovolumic contraction phases (Morley et al., 2007; Wang et al., 2014). With LVAD-induced LV unloading, the RV PV-loop shifted toward larger volumes and higher pressures, indicating that the RV end-diastolic pressure-volume loop was shifting rightward; this is indicative of increased RV diastolic compliance and is a consequence of RV-LV interactions and a leftward septal shift. RV systolic pressure also decreased, also a consequence of RV-LV interactions. In the case of high LVAD operational speed, a secondary “Figure 8” shaped loop is visible at the end of relaxation and the start of passive filling. This ultimately results from the combined effects of RV-LV interactions, pressure-sensitive LVAD operation and the LV being unloaded at a faster rate than it being filled. The degree of unloading that would cause this would likely initiate ventricular arrhythmias in the clinical environment, so data surrounding this type of phenomena is very rare. LVAD speed-dependent septal shifts were clearly evident in the 3-dimensional images. Those images match changes in echocardiographs obtained from LVAD patients at high LVAD speeds, particular in those with right heart failure. For example, Figure 9A shows a patient with low RPMs and normal, right-shifted interventricular septum (traced out by the red line) compared to Figure 9B showing a patient with markedly left-shifted septum. Note the remarkable similarity of these images to those presented in the lower panels of Figures 7A,D.

We found that LVAD support reduced estimates of global LV and, to a lesser extent, septal wall myocardial stresses. However, these improvements were achieved with secondary negative effects on the RV, which experienced a rightward shift toward higher EDPs and larger EDVs with LVAD support, which kept RV stresses high. Additional, potentially negative, effects were seen on the interventricular septum, which showed that LVAD support introduces unnatural bending of the septum with increased localized myofiber stresses. Such deformations are similar to those of a beam undergoing bending deformation, which introduces LVAD speed-dependent regions of tensile stress on the LV side and regions of compressive stress on the RV side of the septal wall (Figure 7). In general, myocardial properties (genetic expression, molecular makeup, structure and function) are modified in response to chronic stresses. However, it is unknown if these abnormal stresses on the myocardium of the septum have any implications for myocardial function as abnormal stresses may relate to the development of right heart failure in the long term.

Over the last few decades, numerical and analytical models of circulatory flow and ventricular assistance have been introduced (e.g., Levin et al., 1995; Vollkron et al., 2002; Morley et al., 2007; Lim et al., 2010). Many of these models represent heart function, without including geometric considerations (often referred to as zero-dimensional models). Despite those limitations, the basic findings derived from such models have generally been in agreement with the present findings. While some research has incorporated functionally realistic ventricular assistance devices, these studies also ignore geometric effects on the heart and, in particular, have not considered ventricular interactions or shifts of septal position (Donahue et al., 2009; Long et al., 2013; Chiu et al., 2014; Selishchev and Telyshev, 2016).

FE modeling enables estimation of regional stress that cannot be measured in patients using alternative techniques. This allows identification of the LVAD speed at which bending and abnormal stresses emerge in the septum. If patient-specific geometries could be incorporated more easily, such modeling could provide interesting metrics with clinical applicability for understanding and perhaps predicting the impact of LVAD speed on septal mechanics.

One goal of developing the present model is to study the degree to which RV-LV interactions and septal shift plays a role in the development of RV failure following LVAD implantation. Many other factors can contribute to the development of RF failure, such as RV myocardial dysfunction, increased pulmonary vascular resistance and volume overload. The impact of those factors, and even RV-LV interactions, are readily ascertained through simpler zero-dimension modeling of the cardiovascular system (HARVI, 2014a,b; Burkhoff et al., 2017). However, it is only through finite element analysis (FEA) that the question of the impact of septal shifts can be determined. For example, although in the present example we demonstrated marked septal shifts at high LVAD speeds accompanied by increased RV EDPs, there were balanced shifts of systolic and diastolic volumes such that cardiac output was mostly preserved, with only minor impact on RV systolic pressure. Thus, in this example, marked septal shift resulted in RV dysfunction and not full-blown failure. However, we have only studied one set of conditions (i.e., one starting RV geometry, one level of myocardial contractility, and one value of pulmonary vascular resistance). A thorough evaluation over a range of conditions and a sensitivity analysis on parameter values is required to fully explore this important question.

## Limitations

The model described in the present study is an improvement over prior models, but several limitations exist. First, the model does not contain atria. Since LV filling dynamics are impacted by atrial contraction, this could have an effect on the diastolic portion of the PV-loop and time-course of change of septal motion. Second, the models of the vascular system are adequate to provide the basic aspects of ventricular afterload and yielded realistic PV-loop shapes, but more sophisticated models that incorporate fluid-structure interactions, the valve geometries and wave reflections would be more accurate. Third, a sensitivity analysis was not performed to quantify the relative impact of material parameters to model results. This was viewed as beyond the scope of this research but should be performed (alongside rigorous validation studies) before computational models contribute to clinical decision making. We intend to address this in our future work. Finally, as noted, we studied only one combination of myocardial properties, RV and LV and vascular properties. Every patient is unique and conclusions arrived at are not generalizable.

## Summary

We described results of an FEA model based on the anatomy of an end-stage failing heart coupled to systemic and pulmonary vascular systems and an LVAD. We demonstrated the anatomic and hemodynamic impact of increasing LVAD speed on global pump function, regional stress distributions, and septal position. Realistic results were obtained in terms of ventricular deformation and PV loops. We demonstrated the expected findings that RV systolic and diastolic properties are affected by LVAD-induced unloading of the LV, resulting in RV dysfunction at high LVAD operating speeds. Having established the foundation of this model, we are poised to address the important question of whether and under what conditions, septal shift, and reduced LV pressure generation are important mechanisms of the development of RV failure following LVAD implantation. The specific conditions studied in the present model demonstrate that septal shifting alone is not sufficient to induce RV failure. Simulations spanning a wide range of conditions are required to fully address this important question.

Beyond this, the methods are generalizable in the sense that patient-specific geometries and vascular properties can be incorporated into the model, with the ultimate purpose of gaining insights into LVAD effects *in vivo*. Such an approach has the potential for predicting hemodynamics, such as the degree of unloading achievable, and the risk of developing right heart failure following LVAD implantation. Advances in patient-specific modeling in other fields of cardiology are already having an impact on clinical practice and it is anticipated that new applications will emerge, especially in the field of heart failure. The methods and results described in the present study have potential to meaningfully advance such efforts.

## Ethics statement

Patient cardiac MR data was obtained as part of the Aliskiren Study in Post-MI Patients to Reduce Remodeling (ASPIRE) trial. The study protocol was written by members of the executive committee of the trial and was approved by the ethics committees at each participating site. Individual patients provided written informed consent in accordance with the Declaration of Helsinki and anonymized data were sent to a core laboratory for analysis.

## Author contributions

KS, TF, DB, and JG were involved in the conception and design of study. KS created the computational models. Acquisition of various data critical to model creation was performed by SS and DB. The analysis and interpretation of modeling results was performed by all authors. KS wrote the first draft of the manuscript. DB and JG wrote sections of the manuscript. All authors contributed to manuscript revision, read and approved the submitted version.

### Conflict of interest statement

The authors declare that the research was conducted in the absence of any commercial or financial relationships that could be construed as a potential conflict of interest.

## Appendix

### Active tension development

The full description of active tension is described by

(A1) $$\begin{array}{l} T_{a}\left(t,l\right)=T_{MAX}\frac{Ca_{0}^{2}}{Ca_{0}^{2}+ECa_{50}^{2}\left(l\right)}\frac{\left(1-\cos\left(\omega \left(t,l\right)\right)\right)}{2} \end{array}$$

where

(A2) $$\begin{array}{l} ECa_{50}\left(l\right)=\frac{C{a_{0}}_{max}}{\sqrt{e^{B\left(l-l_{0}\right)}-1}} \end{array}$$

(A3) $$\omega \left(t,l\right)=\{\begin{array}{cc} \pi \frac{t}{t_{0}}, & \text{when}\;0\leq t\leq t_{0}\\ \pi \frac{t-t_{0}+t_{r}\left(l\right)}{t_{r}}, & \text{when}\;t_{0}\leq t\leq t_{0}+t_{r}\left(l\right)\\ 0, & \text{when}\;t\geq t_{0}+t_{r}\left(l\right) \end{array}$$

(A4) $$\begin{array}{l} t_{r}\left(l\right)\;=\;ml\;+\;b, \end{array}$$

(A5) $$\begin{array}{l} l\;=l_{r}\sqrt{f_{0}\cdot \left(Cf_{0}\right)}, \end{array}$$

with parameters definitions and values provided in Table A1. This mathematical description of active tension ensures a smooth yet steep transition from zero tension at the start of systole to peak active tension, *T*_*max*_, at time *t*_0_ and then a smooth decline back to zero for the specified relaxation time *t*_*r*_.

**Table A1:** Parameter values and definitions for the time-varying elastance constitutive model.

**Active Parameters**	**Value**	**Description**
*t*_0_	120 [ms]	Time to reach peak tension after the initiation of active tension
*m*	1048.9 [s μm−1]	Governs the slope of the relaxation
*b*	−1.7 [s]	Governs the length of relaxation
*l*_0_	1.58 [μm]	The sarcomere length below which no active force develops
*B*	4,750 [μm−1]	Governs the shape of the peak isometric tension-sarcomere length relation
*Ca*_0_	4.35 [μM]	The peak intercellular calcium concentration
*Ca*_0_*max*__	4.35 [μM]	The maximum intercellular calcium concentration
*T*_*max*_	170.0 [kPa]	The maximum active tension able to develop
*l*_*r*_	1.85 [μm]	The initial sarcomere length

### Parameters for lumped circulatory model

All lumped circulatory parameter values are provided in Table A2.

**Table A2:** Parameter values and definitions for the lumped circulatory model.

**Variable**	**Value**	**Description**
R_M_	0.05 [mmHg s ml^−1^]	Impedance due to the mitral valve
R_A_	0.02 [mmHg s ml^−1^]	Impedance due to the aortic valve
R_SYS_	1.80 [mmHg s ml^−1^]	Systemic arterial resistance
R_P_	0.04 [mmHg s ml^−1^]	Impedance due to the pulmonary valve
R_T_	0.02 [mmHg s ml^−1^]	Impedance due to the tricuspid valve
R_PUL_	0.60 [mmHg s ml^−1^]	Pulmonary arterial resistance
C_PA_	8.0 [ml mmHg^−1^]	Compliance of the pulmonary arteries
C_PV_	14.0 [ml mmHg^−1^]	Compliance of the pulmonary veins
C_SA_	2.0 [ml mmHg^−1^]	Compliance of the systemic arteries
C_SV_	38.6 [ml mmHg^1^]	Compliance of the systemic veins
